# Prevalence of vascular disruption anomalies and association with young maternal age: A EUROCAT study to compare the United Kingdom with other European countries

**DOI:** 10.1002/bdr2.2122

**Published:** 2022-11-11

**Authors:** Joan K. Morris, Diana Wellesley, Elizabeth Limb, Jorieke E. H. Bergman, Agnieszka Kinsner‐Ovaskainen, Marie Claude Addor, Jennifer M. Broughan, Clara Cavero‐Carbonell, Carlos M. Dias, Luis‐Javier Echevarría‐González‐de‐Garibay, Miriam Gatt, Martin Haeusler, Ingeborg Barisic, Kari Klungsoyr, Nathalie Lelong, Anna Materna‐Kiryluk, Amanda Neville, Vera Nelen, Mary T. O'Mahony, Isabelle Perthus, Anna Pierini, Judith Rankin, Anke Rissmann, Florence Rouget, Geraldine Sayers, Sarah Stevens, David Tucker, Ester Garne

**Affiliations:** ^1^ St George's University of London London United Kingdom; ^2^ Clinical Genetics, University of Southampton and Wessex Clinical Genetics Service Princess Anne Hospital Southampton United Kingdom; ^3^ University of Groningen, University Medical Center Groningen Department of Genetics Groningen The Netherlands; ^4^ European Commission Joint Research Centre (JRC) Ispra Italy; ^5^ Department of Woman‐Mother‐Child, University Medical Center CHUV Lausanne Switzerland; ^6^ National Disease Registration Service, NHS Digital Leeds United Kingdom; ^7^ Rare Diseases Research Unit Foundation for the Promotion of Health and Biomedical Research in the Valencian Region Valencia Spain; ^8^ Department of Epidemiology Instituto Nacional de Saúde Doutor Ricardo Jorge; Av padre Cruz Lisbon Portugal; ^9^ Ministry of Health of the Basque Government, Directorate for Healthcare Planning Organisation and Evaluation Vitoria‐Gasteiz Spain; ^10^ Directorate for Health Information and Research Malta Congenital Anomalies Registry G'Mangia Malta; ^11^ Medical University of Graz Austria; ^12^ Children's Hospital Zagreb, Centre of Excellence for Reproductive and Regenerative Medicine Medical School University of Zagreb Zagreb Croatia; ^13^ Department of Global Public Health and Primary Care University of Bergen Bergen Norway; ^14^ Division of Mental and Physical Health Norwegian Institute of Public Health Bergen Norway; ^15^ Université de Paris, INSERM U1153, CRESS Obstetrical Perinatal and Pediatric Epidemiology Research Team (EPOPé) Paris France; ^16^ Polish Registry of Congenital Malformations, Department of Medical Genetics Poznan University of Medical Sciences Poznan Poland; ^17^ Center for Clinical and Epidemiological Research University of Ferrara Ferrara Italy; ^18^ Provincial Institute for Hygiene Antwerp Belgium; ^19^ Department of Public Health Health Service Executive‐South Cork Ireland; ^20^ Auvergne Registry of Congenital Anomalies (CEMC‐Auvergne), Department of Clinical Genetics, Centre de Référence des Maladies Rares University Hospital of Clermont‐Ferrand, CNRS‐UMR 6602, Institut Pascal, Axe TGI, équipe PEPRADE Clermont‐Ferrand France; ^21^ Unit of Epidemiology of Rare Diseases and Congenital Anomalies, Institute of Clinical Physiology National Research Council Pisa Italy; ^22^ Fondazione Toscana Gabriele Monasterio Pisa Italy; ^23^ Population Health Sciences Institute, Faculty of Medical Sciences Newcastle University Newcastle upon Tyne United Kingdom; ^24^ Malformation Monitoring Centre Saxony‐Anhalt Medical Faculty Otto‐von‐Guericke‐University Magdeburg Magdeburg Germany; ^25^ Brittany Registry of Congenital Anomalies, CHU Rennes, Univ Rennes, Inserm, EHESP Irset (Institut de recherche en santé, environnement et travail) ‐ UMR_S 1085 Rennes France; ^26^ Health Intelligence Research and Development Health Service Executive Dublin Ireland; ^27^ National Disease Registration Service NHS Digital Leeds United Kingdom; ^28^ Public Health Knowledge and Research, Public Health Wales Singleton Hospital Swansea United Kingdom; ^29^ Department of Paediatrics and Adolescent Medicine, Lillebaelt Hospital University Hospital of Southern Denmark Kolding Denmark

**Keywords:** anomalies, congenital, disruption, surveillance, vascular

## Abstract

**Background:**

Younger mothers are at a greater risk of having a pregnancy with gastroschisis and the risk is higher in the United Kingdom than other European countries. Gastroschisis is thought to be a vascular disruption anomaly and the aim of this study was to analyze the prevalence of other possible vascular disruption anomalies to determine whether both the younger maternal age and the UK associations also occur with these anomalies.

**Methods:**

All pregnancies with anomalies considered potentially due to vascular disruption from January 1, 2005 to December 31, 2017 from 26 European population‐based congenital anomaly registries who were members of EUROCAT were analyzed. Multilevel models were used to allow for differences between registries when analyzing associations with maternal age, year of birth and whether the registry was in the United Kingdom.

**Results:**

There were 5,220 cases with potential vascular disruption anomalies, excluding chromosomal and genetic conditions, with a prevalence of 8.85 per 10,000 births in the United Kingdom and 5.44 in the other European countries. The prevalence per 10,000 births of gastroschisis (4.45 vs. 1.56) and congenital constriction bands (0.83 vs. 0.42) was significantly higher in the United Kingdom, even after adjusting for maternal age. However, transverse limb reduction defects had a similar prevalence (2.16 vs. 2.14 per 10,000). The expected increased prevalence in younger mothers was observed for vascular disruption anomalies overall and for the individual anomalies: gastroschisis and congenital constriction bands.

**Conclusion:**

Vascular disruption anomalies that had an increased risk for younger mothers (such as gastroschisis) had a higher maternal age standardized prevalence in the United Kingdom, while vascular disruption anomalies with weaker associations with younger mothers (such as transverse limb reduction defects) did not have an increased prevalence in the United Kingdom, which may indicate a different etiology for these anomalies.

## 1 INTRODUCTION

The reduction in blood flow to a structure in the fetus that has already developed normally causes hypoxia leading to cell damage and tissue loss. After the event, there may be residual tissue loss and structural abnormalities (Holmes, Westgate, Nasri, & Toufaily, 2018). Several congenital anomalies, including gastroschisis, have been attributed to the occurrence of a vascular disruption event during pregnancy (Hume et al., 1997; van Gelder, van Rooij, de Jong‐van den Berg, & Roeleveld, 2014). Previous studies have shown that younger mothers are at a greater risk of having a pregnancy with gastroschisis and also that the maternal age adjusted prevalence is higher in the United Kingdom than in other European countries (Baldacci et al., 2020; Feldkamp et al., 2019; Loane, Dolk, Morris, & Group, 2009; Rittler et al., 2015). It is not known whether these findings also apply to other anomalies likely to be due to vascular disruption.

EUROCAT is a European network of population‐based registries for the epidemiologic surveillance of congenital anomalies (https://eu-rd-platform.jrc.ec.europa.eu/eurocat_en) (Kinsner‐Ovaskainen et al., 2018; Tucker et al., 2018). The aim of this study was to analyze anomalies, other than gastroschisis, that are likely to be due to vascular disruption to determine if younger maternal age and being born in the United Kingdom are associated with an increased risk of these anomalies.

## 2 METHODS

The methods of data collection in EUROCAT registries have been described previously in detail (Boyd et al., 2011). In brief, congenital anomaly registries use active case finding and passive notification, such as hospital discharge diagnoses, birth and death certificates and post mortem examinations to identify cases with congenital anomalies. All major structural congenital anomalies, syndromes and chromosomal anomalies occurring in livebirths (LB), fetal deaths (FD) with a gestational age (GA) ≥ 20 weeks and terminations of pregnancy after prenatal diagnosis of congenital anomaly (TOPFA) at any gestation are included in the database. Minor anomalies are excluded based on a list of ICD10 codes for exclusion in EUROCAT Guide 1.4 (EUROCAT Guide 1.4). In this study, all congenital anomalies have been coded according to ICD10 with the British Paediatric Association (BPA) extension for further specification.

All full member registries were invited to take part in the study and 26 registries including six from the United Kingdom agreed to participate. Data were extracted from the EUROCAT database on October 23, 2019 (Table 1). All birth outcomes (LB, FD, and TOPFA) with an ICD10 code indicating an anomaly due to vascular disruption, as defined by the EUROCAT Coding and Classification Committee, born from January 1, 2005 to December 31, 2017 were included. Not all registries contributed data for all 13 years. Data about each case included year of birth, type of birth, GA at birth or termination, birthweight, sex, time of diagnosis, maternal age, and associated congenital anomalies.

**TABLE 1:** Number of cases and prevalence of cases with vascular disruption anomalies (excluding genetic conditions) according to registry for birth years 2005–2017

Registry	First year	Last year	Vascular disruption cases	Total births (10,000 s)	Prevalence per 10,000 births (95% CI)	Percentage of all births to mothers <20 years old (%)
Denmark, Odense	2005	2015	63	5.5	11.5 (8.9–14.7)	1.6
UK, Wales	2005	2017	475	44.6	10.7 (9.7–11.7)	6.9
UK, PHE/Northern England	2005	2017	432	42.5	10.2 (9.2–11.2)	7.6
Germany, Saxony Anhalt	2005	2017	216	22.5	9.6 (8.3–10.9)	4.9
UK, PHE/Wessex	2005	2017	353	38.8	9.1 (8.2–10.1)	4.8
UK, PHE/EMSY	2005	2017	631	73.1	8.6 (8.0–9.3)	6.7
UK, PHE/SW England	2005	2017	544	64.2	8.5 (7.8–9.2)	4.9
France, Auvergne	2005	2017	146	17.4	8.4 (7.1–9.9)	2.5
France, Paris	2005	2017	232	34	6.8 (6.0–7.8)	0.8
The Netherlands, North	2005	2017	148	22	6.7 (5.7–7.9)	1.5
Switzerland, Vaud	2005	2017	66	10.5	6.3 (4.9–8.0)	0.7
UK, PHE/Thames Valley	2005	2017	236	38.8	6.1 (5.3–6.9)	3.3
Spain, Basque	2005	2016	145	24.2	6.0 (5.1–7.0)	1.3
France, Brittany	2011	2017	144	24.5	5.9 (5.0–6.9)	1.1
Croatia, Zagreb	2005	2017	46	8.3	5.5 (4.0–7.4)	3.8
Ireland, Cork & Kerry	2005	2017	68	12.5	5.4 (4.2–6.9)	2.0
Poland, Wielkopolska	2005	2017	258	49.9	5.2 (4.6–5.8)	4.0
Belgium, Antwerp	2005	2016	126	24.5	5.1 (4.3–6.1)	1.8
Italy, Emilia Romagna	2005	2017	248	50.5	4.9 (4.3–5.6)	1.3
Austria, Styria	2005	2016	61	12.6	4.8 (3.7–6.2)	2.9
Italy, Tuscany	2005	2017	178	37.9	4.7 (4.0–5.4)	1.2
Ireland, SE Ireland	2005	2016	41	8.8	4.7 (3.4–6.3)	3.2
Ireland, Dublin	2005	2012	95	21	4.5 (3.7–5.5)	3.3
Malta, Malta	2005	2016	20	5	4.0 (2.4–6.2)	5.1
Spain, Valencian Region	2007	2017	175	53	3.3 (2.8–3.8)	2.4
Portugal, South Portugal	2005	2017	73	24.1	3.0 (2.4–3.8)	4.5
Total			5,220	770.7		

Abbreviations: EMSY, East Midlands and South Yorkshire; PHE, Public Health England; SW, South West.

## 3 CLASSIFICATION OF VASCULAR DISRUPTION ANOMALIES

Cases with genetic syndromes or microdeletions, skeletal dysplasia, genetic skin disorders, or chromosomal anomaly were excluded from the analysis. The EUROCAT Coding and Classification Committee, based on the papers by Hume et al. (1997) and van Gelder et al. (2014), identified the following anomalies as likely to be due to vascular disruption: gastroschisis; transverse limb reduction defects; complete absence of a limb; absence, atresia and stenosis of small intestine; congenital constriction bands, limb–body‐wall complex, hydranencephaly, Poland's anomaly, and Moebius syndrome (Hume et al., 1997; van Gelder et al., 2014). As for all these anomalies, the size of the contribution of vascular disruption to the etiology is under debate, it was decided to include as comprehensive a list as possible and present results for specific anomalies to enable the reader to interpret the results for themselves.

All cases were reviewed and classified into one diagnostic group of potential vascular disruption cases. Cases with Moebius anomaly (Q8706), Poland anomaly (Q7982), congenital constriction bands (Q7980) and limb–body‐wall complex (Q795) were classified into these groups irrespective of associated anomalies. For the four structural anomaly diagnostic groups (hydranencephaly [Q0435]; gastroschisis [Q793]; small intestinal atresia without gastroschisis [Q411, Q412, Q418 without Q793]; and limb anomalies including complete absence of a limb [Q710, Q720, Q730]) and transverse limb reduction defects (Q712, Q713, Q722, Q723), there were only a few cases with more than one of these anomalies, except for gastroschisis (Q793) and small intestinal atresia (Q411, Q412,Q418), which is classified by EUROCAT as gastroschisis (sequence).

An additional category “transverse limb reduction defect with gastroschisis or small intestinal atresia” was also defined due to there being a significant number of cases with both anomalies. All cases were reviewed by a geneticist (D.W.) and a pediatrician (E.G.) with the text fields providing additional information to the ICD10 codes. As the code Q795 for limb–body‐wall complex is not specific for that anomaly, the written text description for the cases was reviewed. Cases were included if there was text for LBWC/body stalk anomaly or if text for abdominal wall defect was associated with anomaly codes and written text for anencephaly/encephalocele and/or limb defects.

## 4 STATISTICAL METHODS

Multilevel generalized linear models were fitted specifying a binomial distribution with a logit link to analyze the effect of maternal age and birth year on the prevalence of vascular disruption anomalies. Registries were modeled as random effects. Maternal age was analyzed as a categorical variable in the following age groups: <20, 20–24, 25–29, 30–34, 35–39, and 40+ in order to allow a nonlinear association with age to be modeled.

## 5 RESULTS

**FIGURE 1:**
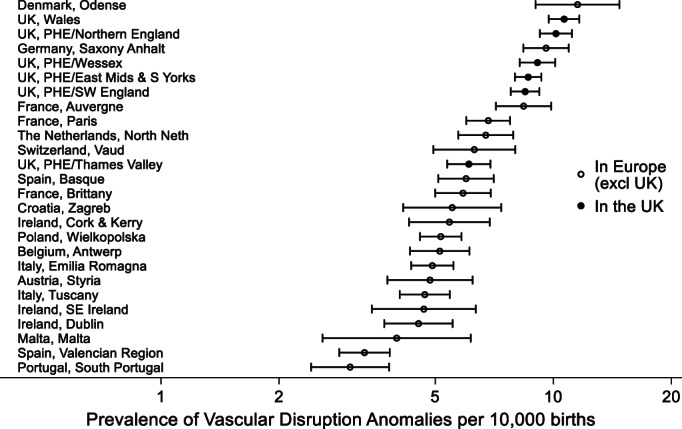
Prevalence of vascular disruption in EUROCAT registries (2005–2017) with 95% CIs

There were 5,220 cases with vascular disruption anomalies excluding chromosomal and genetic conditions with a prevalence of 8.85 per 10,000 births in the United Kingdom and 5.44 per 10,000 births in other European countries. The prevalence varied considerably across European congenital anomaly registries (see Figure 1 and Table 1). There appeared to be a higher prevalence in the registries from the United Kingdom compared to those from Europe, with five of the seven registries with the highest prevalence being from the United Kingdom.

Table 2 shows that the types of anomalies that were classified as likely to be due to a vascular disruption differed between the UK registries and those from Europe with gastroschisis accounting for 50% of all cases in the United Kingdom and only 29% elsewhere and the prevalence being 4.45 in the United Kingdom compared to 1.56 per 10,000 births elsewhere. A similar pattern occurred with congenital constriction bands and limb–body‐wall complex being more prevalent in the United Kingdom. However, both transverse limb reduction defects and small intestinal atresia had similar prevalences in the United Kingdom and Europe (2.16 vs. 2.14 and 0.85 vs. 0.86, respectively). Gastroschisis and congenital constriction bands were significantly associated with younger maternal ages. Anomalies with higher prevalence rates in the United Kingdom tended to be more associated with younger maternal age (*p* = .06 Spearman's correlation of relative odds per 5 year increase in age with odds born in the United Kingdom).

**TABLE 2:** Type of anomaly according to whether the case was born in the United Kingdom or in other European countries

	In Europe (excl. UK)				In the United Kingdom			Prevalence in Europe (excl. UK) versus in the United Kingdom^a^
	Number of cases	%	Prevalence per 10,000 births^a^	Risk reduction per 5 years increase in maternal age^a^	Number of cases	%	Prevalence per 10,000 births^a^	
Transverse limb reduction defects	1,004	39	2.14	0.94 (0.89–1.00)	653	24	2.16	*p* = .921
Gastroschisis	730	29	1.56	0.46 (0.43–0.49)	1,345	50	4.45	*p* < .001
Small intestinal atresia	401	16	0.86	1.05 (0.96–1.15)	258	10	0.85	*p* = .681
Congenital constriction bands	196	8	0.42	0.86 (0.76–0.98)	251	9	0.83	*p* = .054
Poland's anomaly	81	3	0.17	0.95 (0.78–1.16)	29	1	0.10	*p* = .090
Limb–body‐wall complex	58	2	0.12	0.89 (0.70–1.13)	103	4	0.34	*p* = .085
Hydranencephaly	38	1	0.08	0.86 (0.65–1.14)	20	1	0.07	*p* = .486
Moebius syndrome	31	1	0.07	1.10 (0.79–1.52)	7	0	0.02	*p* = .024
Transverse limb reduction defect with gastroschisis or intestinal atresia	10	0	0.02	^b^	5	0	0.02	*p* = 1.00
Total	2,549	100	5.44	0.77 (0.74–0.80)	2,671	100	8.85	*p* < .013

^a^ Adjusted for registry and maternal age.

b Model not fitted due to lack of data.

Table 3 highlights differences in cases between the United Kingdom and the other European countries, with mothers in the United Kingdom being more likely to be younger with 19% under 20 years of age compared with 6% in other European countries. The proportions of cases detected prenatally were higher in the United Kingdom (69% vs. 59%). The live born babies were more likely to be singletons and around 100 g lighter (not a clinically important result).

**TABLE 3:** Characteristics of the cases with vascular disruption anomalies

	In Europe (excl. UK)		In the United Kingdom		Comparison in Europe (excl. UK) versus in the United Kingdom
	Number cases	%^a^	Number cases	%	
Livebirth	2,056	81	2,118	79	*p* = .07
Fetal death	75	3	110	4	
TOPFA	417	16	443	17	
Male	1,295	53	1,364	53	*p* = .62
Female	1,149	47	1,145	46	
Indeterminate sex	8	0	8	0	
Missing	97		154		
Singleton	2,415	95	2,553	96	*p* < .001
Multiple	129	5	79	4	
Missing	5		39		
When Discovered					
At_birth	862	36	341	26	*p* < .001
After_birth	124	5	159	5	
Prenatal	1,423	59	2,075	69	
Missing	140		96		
Gestational age at live birth					
<37 weeks	808	40	869	42	*p* = .37
37–41 weeks	1,201	59	1,212	58	
≥42 weeks	12	1	21	1	
Missing	35		16		
Maternal age (years)					
< 20	155	6	500	19	*p* < .001
20–24	498	20	840	31	
25–29	669	27	629	24	
30–34	685	27	466	17	
35–39	410	16	182	7	
40+	106	4	47	2	
Missing	26		7		
Birthweight in live births (kg)					
< 37 weeks	2.15 (2.11–2.19)		2.15 (2.11–2.18)		*p* < .001
37–41 weeks	3.09 (3.06–3.12)		2.95 (2.92–2.99)		
≥42 weeks	3.60 (3.46–3.73)		3.39 (3.11–3.67)		

a Percentage of nonmissing values.

**FIGURE 2:**
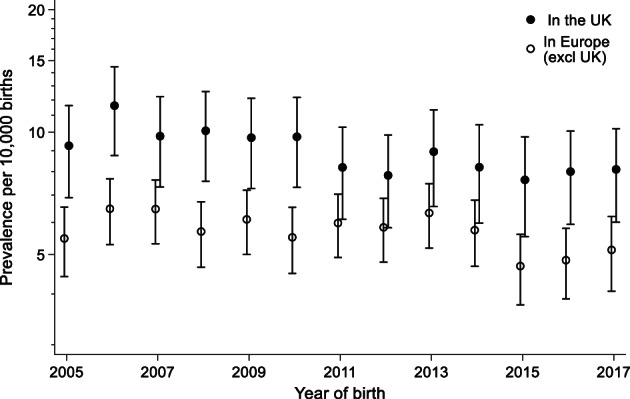
Prevalence of vascular disruption in EUROCAT registries from 2005 to 2017 (adjusted for registry) with 95% CIs

**FIGURE 3:**
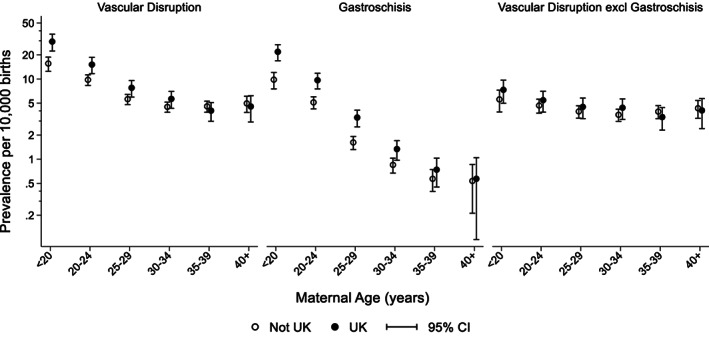
Prevalence of vascular disruption including gastroschisis, gastroschisis only and vascular disruption excluding gastroschisis according to maternal age and whether born in the United Kingdom or in other European countries (adjusted for registry) with 95% CIs

**FIGURE 4:**
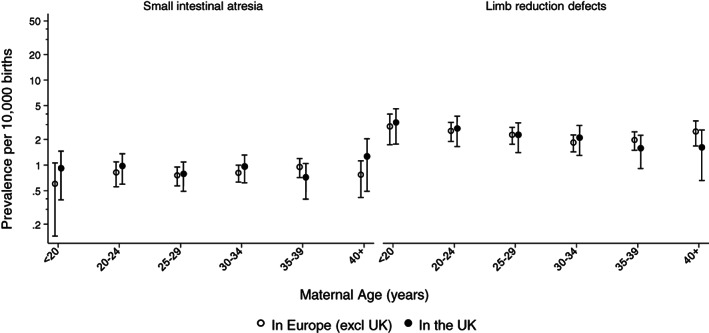
Prevalence of small intestinal atresia and transverse limb reduction defects according to maternal age and whether born in the United Kingdom or in other European countries (adjusted for registry) with 95% CIs

Figure 2 shows that the prevalence of vascular disruption anomalies decreased slightly from 2005 to 2017, with the decrease being similar in United Kingdom and the other European countries. Figure 3 shows that overall there is a strong association of a vascular disruption anomaly with maternal age. Younger mothers are at an increased risk and the risk is also higher in the United Kingdom than in the other European countries for the younger mothers. The association with age and with being born in the United Kingdom is much stronger for gastroschisis, but a slight decreasing risk with age does remain after cases with gastroschisis are excluded. Figure 4 shows that vascular disruption anomalies that are not as strongly associated with low maternal age (e.g., small intestinal atresia and transverse limb reduction defects) have a similar prevalence in the United Kingdom and the other European countries.

## 6 DISCUSSION

This study has shown that the prevalence of vascular disruption anomalies is higher in the United Kingdom than in the other European countries in EUROCAT. Part of this is explained by the fact that 5.3% of mothers were under age 20 in the United Kingdom compared to only 2.3% in the other European countries. However, younger mothers in the United Kingdom compared with mothers of a similar age in the other European countries, were at a higher risk for those vascular disruption anomalies with stronger associations with young maternal age, such as gastroschisis. Anomalies with no increased risk for younger mothers did not have an increased risk in the United Kingdom compared to Europe, such as small intestinal atresia. A study on small intestinal atresia using an earlier birth cohort from EUROCAT (1990–2006), in agreement with our study, found no increased prevalence for all small intestinal atresia in the United Kingdom (Best et al., 2012).

Denmark appeared to have the highest prevalence of these anomalies, but the region covered by the registry is small (around 5,000 births per year) and the confidence intervals are wide (see Table 1). The most frequent vascular disruption anomaly in this registry was transverse limb reduction defects (39%), which has a weak association with young maternal age (see Figure 4).

In addition to gastroschisis, congenital constriction bands and limb–body‐wall complex (Lowry, Bedard, & Sibbald, 2017; Syvänen et al., 2021) have also been associated with younger maternal ages, although not consistently (Martínez‐Frías, Bermejo, & Rodríguez‐Pinilla, 2000; Werler, Bosco, & Shapira, 2009). Teenage mothers have also been found to be at a significantly greater risk of maternal infection syndromes, tricuspid atresia, anencephalus, nervous system and digestive system anomalies (Loane et al., 2009). Loane et al. did not observe an increased risk for younger mothers in the United Kingdom compared with other European countries for these anomalies, but this may be due to the fact that only larger more heterogeneous anomaly groups were analyzed with no analysis restricted to vascular disruption anomalies (Best et al., 2012).

Several studies have found that septo‐optic dysplasia is associated with younger maternal ages (Atapattu et al., 2012; Garne et al., 2018; Khaper et al., 2017). However, a recent EUROCAT study found that there was no indication of a higher prevalence in the United Kingdom compared with continental Europe, which indicates that the link of younger maternal ages in the United Kingdom may be specific to vascular disruption anomalies.

The strength of this study is that it includes population‐based data from 26 congenital anomaly registries in Europe, all of whom are members of EUROCAT. All registries use the same inclusion criteria for major anomalies and the same coding and classification system for congenital anomalies enabling valid comparisons across Europe to be made. A limitation is the varying level of ascertainment for the different registries, which may be influenced by the availability of prenatal diagnosis and uptake of TOPFA in different countries. Despite general coding recommendations from EUROCAT, the coding of transverse limb reduction defects may differ across registries. Both congenital constriction bands and limb–body‐wall complex may be more readily diagnosed in the United Kingdom compared with continental Europe. In addition, whether constriction bands are mentioned on a physical exam could vary widely depending on their size, location, and the degree of detail included the record. However, gastroschisis is unlikely to be under ascertained in any registry.

Cigarette smoking, alcohol consumption, recreational drug use, stress, and a diet lacking in fruit and vegetables have been identified as risk factors for gastroschisis and it may be that younger mothers in the United Kingdom are more exposed to them than younger mothers in other European countries (Caspers et al., 2013; Draper et al., 2008; Palmer et al., 2013; Paranjothy et al., 2012). However, a study of maternal smoking during pregnancy in 2011/2012 did not find that more women smoked during pregnancy in the United Kingdom compared with other European countries (Smedberg, Lupattelli, Mårdby, & Nordeng, 2014). This suggests that other risk factors that were not investigated in this report, alone or in combination with young maternal age, could contribute to the development of vascular disruption anomalies.

In summary, vascular disruption anomalies that had an increased risk for younger mothers (such as gastroschisis) had a higher maternal age standardized prevalence in the United Kingdom, while vascular disruption anomalies with weaker associations with younger mothers (such as transverse limb reduction defects) did not have an increased prevalence in the United Kingdom, which may indicate a different etiology for these anomalies.

## Data Availability

The data that support the findings of this study are available on request from the corresponding author. The data are not publicly available due to privacy or ethical restrictions.
